# Metabolomics Analysis Reveals Tissue-Specific Metabolite Compositions in Leaf Blade and Traps of Carnivorous *Nepenthes* Plants

**DOI:** 10.3390/ijms21124376

**Published:** 2020-06-19

**Authors:** Alberto Dávila-Lara, Carlos E. Rodríguez-López, Sarah E. O’Connor, Axel Mithöfer

**Affiliations:** 1 Research Group Plant Defense Physiology, Max Planck Institute for Chemical Ecology, 07745 Jena, Germany; adavila-lara@ice.mpg.de; 2 Departamento de Biología, Universidad Nacional Autónoma de Nicaragua-León (UNAN), 21000 León, Nicaragua; 3 Department of Natural Product Biosynthesis, Max Planck Institute for Chemical Ecology, 07745 Jena, Germany; clopez@ice.mpg.de (C.E.R.-L.); oconnor@ice.mpg.de (S.E.O.)

**Keywords:** *Nepenthes*, carnivorous plants, UPLC-qToF-MS, metabolomics, tissue specificity, cheminformatics

## Abstract

*Nepenthes* is a genus of carnivorous plants that evolved a pitfall trap, the pitcher, to catch and digest insect prey to obtain additional nutrients. Each pitcher is part of the whole leaf, together with a leaf blade. These two completely different parts of the same organ were studied separately in a non-targeted metabolomics approach in *Nepenthes x ventrata,* a robust natural hybrid. The first aim was the analysis and profiling of small (50–1000 *m*/*z*) polar and non-polar molecules to find a characteristic metabolite pattern for the particular tissues. Second, the impact of insect feeding on the metabolome of the pitcher and leaf blade was studied. Using UPLC-ESI-qTOF and cheminformatics, about 2000 features (MS/MS events) were detected in the two tissues. They showed a huge chemical diversity, harboring classes of chemical substances that significantly discriminate these tissues. Among the common constituents of *N. x ventrata* are phenolics, flavonoids and naphthoquinones, namely plumbagin, a characteristic compound for carnivorous Nepenthales, and many yet-unknown compounds. Upon insect feeding, only in pitchers in the polar compounds fraction, small but significant differences could be detected. By further integrating information with cheminformatics approaches, we provide and discuss evidence that the metabolite composition of the tissues can point to their function.

## 1. Introduction

**Figure 1:**
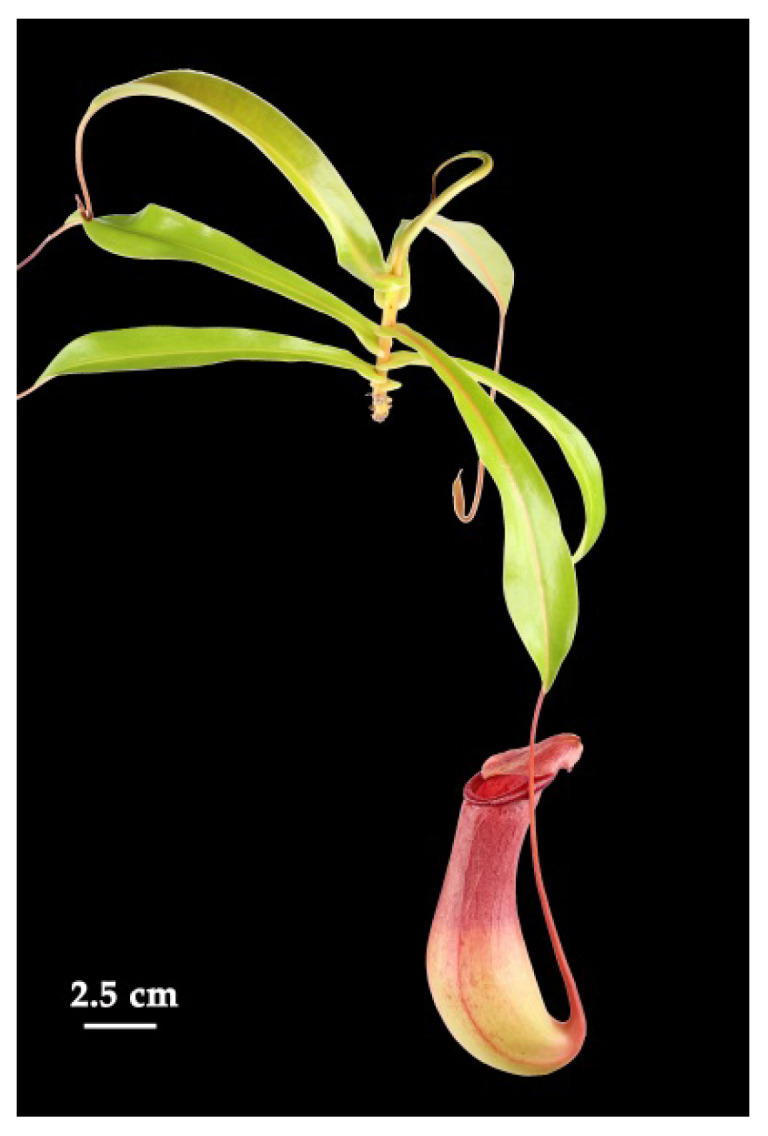
*Nepenthes x ventrata*. Natural hybrid of *N. ventricosa* and *N. alata*.

Metamorphosis of plant organs is a common feature in higher plants and often an adaptation to the particular environment. Metamorphosis covers genetically fixed changes in both morphology and anatomy leading to new structural or functional modifications. In higher plants, leaves are mainly involved in photosynthesis and transpiration, but many leaf metamorphoses are also known for exhibiting new functions. Examples are spines as protection against herbivores (cacti), needles to reduce water loss (conifers), bulbs for storage of water and nutrients (onion), and tendrils for climbing (pea). Striking structures of leaf metamorphosis are found in many carnivorous plants that live on nutrient-poor soil and catch animal prey to get additional nutrients, such as nitrogen and phosphate [1,2]. Here, the leaves are employed in catching prey, mainly insects. For instance, in Venus flytrap (*Dionaea muscipula*), rapidly closing snap traps are found, in sundew (*Drosera*) species sticky flypaper traps, and in bladderwort (*Utricularia)* species sucking bladder traps [1,2]. Another type of trap is realized in so-called pitcher traps that can be found in the genus *Nepenthes* (Figure 1), occurring in Southeast Asia. 

**Figure 2:**
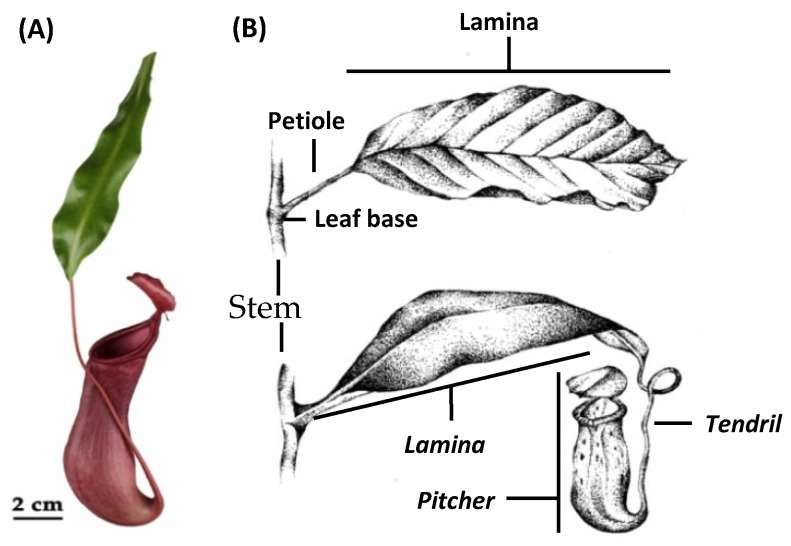
Comparison of leaf morphology. (**A**) *Nepenthes x ventrata* leaf. (**B**) Typical foliage leaves (upper), *Nepenthes* leaf (below). In italics, the leaf parts developed in *Nepenthes* as result of metamorphosis of the typical leaf parts. For further explanation, see the text. Copyright © of drawing (**B**) held by Sarah Zunk.

These passive traps attract prey to the pitcher opening, the peristome, which is extremely slippery for insects causing them to fall into the pitcher. The lower part of the pitcher is filled with a fluid where the prey drowns. Subsequently, plant-derived hydrolytic enzymes inside the fluid digest the prey and generate absorbable forms of nutrients, which are taken up and delivered further to the plant body through bi-functional glands [2,3]. In *Nepenthes* species, the whole leaf underwent an extensive metamorphosis: the typical leaf lamina (synonym: leaf blade) turned into a pitcher for catching prey, the petiole into a tendril to climb, and the leaf base into a basal leaf-derived leaf blade (from now on: leaf blade) substituting the lamina to ensure photosynthesis (Figure 2) [4,5]. 

For many years, scientists studied the different trapping mechanisms in order to understand their function and biomechanics. However, changes and adaptations in leaf morphology and anatomy also come along with changes in the physiology, biochemistry, and molecular biology of carnivorous plants. Thus, in recent years, many studies in carnivorous plants focused more and more on molecular aspects and “omics” approaches, except metabolomics. Those studies have produced more and deeper insights in the molecular events accompanying the various steps necessary for successful prey hunting and digestion, suggesting, for example, that plant carnivory originates from defense mechanisms [6,7,8,9,10,11,12]; however, most studies are still related to the particular traps. 

In *Nepenthes*, the pitcher fluid was investigated in detail, including its proteome [13,14,15] and the composition of organic and inorganic low-molecular-weight compounds [16]. Based on such studies, we learned that the pitcher fluids consist of enzymes necessary for digestion and also defensive proteins belonging to the group of pathogenesis-related proteins [17]. Moreover, the pitcher fluid is poor in inorganic nutrients and contains secondary metabolites with antimicrobial properties, i.e., naphthoquinones; droserone and 5-*O*-methyl droserone are described for *N. khasiana* [18] and plumbagin and 7-methyl-juglon for *N. ventricosa* [16]. These compounds are not widespread in plants but very often occur in carnivorous plants of the order Nepenthales [19], a *sensu stricto* sister group to Caryophyllales [5]. For *Nepenthes*, some of these naphthoquinones were described as inducible by chitin and prey [18,20], suggesting a functional role after prey catch. Naphthoquinones are highly bioactive compounds with defense-related properties [21]. Therefore, it has for a long time been suggested that these compounds are involved in protection against various microbes and pest attack and preserving prey during digestion [16,17,18,19]. Plumbagin and some other naphthoquinone derivatives have also been found in various tissues of *Nepenthes* species including the pitchers [16,20,22,23]. In addition, in the literature, the presence of carotenoids, flavonoids, sterols and triterpenes was mentioned for *Nepenthes* leaves [2,24,25]. 

As many carnivorous plants, including *Nepenthes*, harbor a huge chemical diversity, many secondary metabolites from carnivorous plants are currently isolated for pharmaceutical, biotechnological and pseudo-medical use [2,26,27]. This approach *per se* has led to pharmacologically valuable molecules, and, notably in times of an ongoing pandemic, its value is obvious. However, metabolomics studies to better understand the role of metabolites concerning their ecological function in a carnivorous plant are not available but nevertheless important. As suggested by Hatcher and colleagues [19], the metabolite diversity may represent a mechanism supporting the evolution of carnivory and the ability to cope with new and harsh environments. In addition, regarding the metabolome, carnivorous plants’ responses to the assimilation of animal-derived nutrients remain largely unknown. Thus, the examinations of metabolite changes in pitcher and leaf blade tissues before and after prey digestion may also provide insight into dynamic processes in plant metabolism. 

In order to address these questions, we used a non-targeted approach to analyze and compare, in *Nepenthes x ventrata*, the ionizable metabolites of specialized tissues; i.e., pitcher traps that are involved in prey catch and (basal) leaf blades involved in photosynthesis. In addition, we analyzed changes in the metabolite composition upon insect prey digestion. Besides these ecological aspects, the unique metamorphosis of a typical leaf organ into highly specialized tissues adds a fascinating developmental aspect. 

## 2. Results

### 2.1. Metabolomics Reveals a Loss in Metabolite Load and Diversity in the Specialized Pitcher Organ

*Drosophila melanogaster*-fed and non-fed pitchers and related leaf blades of *N. x ventrata* were subject to independent polar and non-polar extractions. Extracts were analyzed by UPLC-ESI-qTOF in positive mode, with data-dependent fragmentation. Data was acquired in positive mode due to higher sensitivity and the higher quality of fingerprint predictions of SIRIUS+CIS-FingerID in positive as compared to negative mode. Since, in polar extractions, the chromatograms were dominated by a few peaks, to increase the coverage the samples were injected twice; as concentrated extracts and as ten-fold dilution. Using MetaboScape^®^, in the non-polar extraction 1396 peaks were detected and adducts grouped into 1226 features, 984 of which had at least one MS/MS event. In the polar extracts, 1398 and 560 peaks were detected, grouped in 1250 and 509 features, with 1012 and 383 fragmentation events in concentrated and diluted samples, respectively; both matrices of polar features were concatenated. 

**Figure 3:**
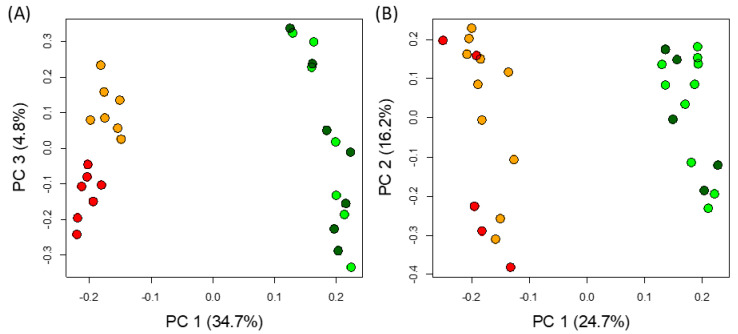
Unsupervised analysis of all detected features. PCA analysis of features detected in polar (**A**) and non-polar (**B**) extracts. Tissue and feeding status are indicated by the colors dark green and light green, showing fed and not-fed leaves, and red and orange, showing fed and not-fed pitchers, respectively.

To gain an overview of the metabolomics changes, non-supervised analysis was performed separately on both polar and non-polar extracts. For both extractions, a Principal Component Analysis (PCA) showed that the main source of variation is the tissue, separated by the first component, explaining 35% and 25% of the variance in polar and non-polar metabolites, respectively (Figure 3). Interestingly, only the polar features of fed and non-fed pitchers were separated in the PCA (by the third component), explaining around 5% of the variance (Figure 3a). None of the other combinations of PCs, cumulatively explaining up to 95% of the variance, managed to separate samples by feeding status. Remarkably, a consistent trend can be seen in the score plots (Figure S1), where leaf-specific features have a higher *m*/*z* than pitcher-specific peaks in both polar and non-polar extracts. 

**Figure 4:**
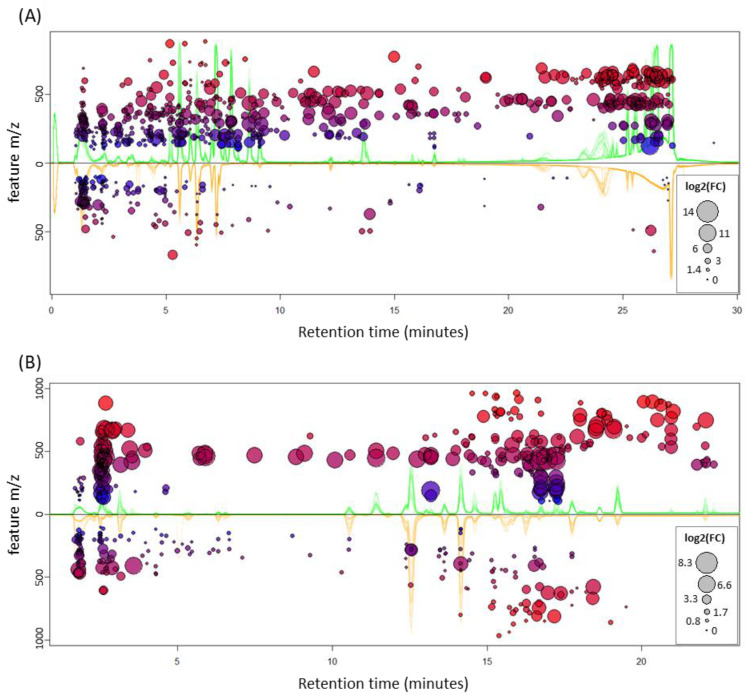
Mirror plots of differentially accumulated features (DAFs). DAFs (FDR < 0.01) in polar (**A**) and non-polar (**B**) extracts are shown for leaf (top) and pitcher (bottom). Circle size depicts the absolute value of the log_2_ of the average fold change, on the top if it is over-accumulated in leaf, and on the bottom otherwise. Color and y-axis value depict the *m*/*z* value of the feature, with blue being low- (100) and red high- (1000) *m*/*z* features; the further away from the origin, the higher the *m*/*z*, as indicated by the y-axis. The superimposed, raw base-peak chromatograms (BPC) of all runs are shown in the background, colored accordingly: green, all leaf BPCs; orange, all pitcher BPCs.

To complement the non-supervised analysis and to estimate the effect of tissue type and feeding status, two-way ANOVA tests were run on the features. Ratifying the previous observation, only tissue had features that were significantly different (FDR < 0.01). After removing duplicated signals, in the polar fraction 797 differentially accumulated features (DAFs) were found, with the vast majority (634) being highly accumulated in leaf compared to pitcher (163 features; Figure S2). Correspondingly, the non-polar fraction had 449 DAFs that were more balanced, with 272 and 177 over-accumulated in leaf and pitcher, respectively (Figure S3). The DAFs are shown in the cloud plot of Figure 4, where the trend hinted at by the PCA score plots is confirmed: in both polar and non-polar extracts, features over-accumulated in leaf are of higher *m*/*z* than those over-accumulated in pitcher, with a difference of medians of 122 Th and 121 Th, respectively (Figure S4).

Moreover, besides the finding that leaves show more significantly accumulated features, the fold-change of those features is also remarkably higher (size of the circles in Figure 4) than the features over-accumulated in pitchers (Figure S5).

### 2.2. Database-Independent Spectral Analysis Identifies Key Substructures in DAF

Assignation of feature identity is a complicated endeavor, which in MS-based metabolomics relies heavily on compound databases. Unequivocal identification of a compound requires isolation and analysis by NMR, and putative identification by fragmentation patterns requires manual curation of candidate lists, generated by algorithms that automate comparisons to databases. Given that *Nepenthes* is an understudied genus, we expect few of the detected compounds to be present in chemical databases; however, some structural information can be directly extracted from the MS/MS spectra. 

With that purpose, for every adduct of all DAFs, we collected fragmentation spectra and analyzed it using SIRIUS [28,29,30] and CSI-FingerID [31], from which the best-predicted fingerprint vectors for each DAF were selected for analysis. In total, 580 DAFs (72%) from the polar and 212 DAFs (47%) from the non-polar fractions were each assigned a vector of chemical fingerprints. For reference, only 11 DAFs (2%) of the non-polar fraction had a hit using the extended database LipidBlast [32]. CSI-FingerID vectors contain 2937 chemical fingerprints [31] to which we assigned one of three values (present, absent, and uncertain) based on their posterior probabilities. We then calculated enrichment probabilities of the presence and absence of each fingerprint in each tissue, separately for polar and non-polar; the significantly enriched ones (FDR < 0.05) are shown in Tables S1 and S2.

**Figure 5:**
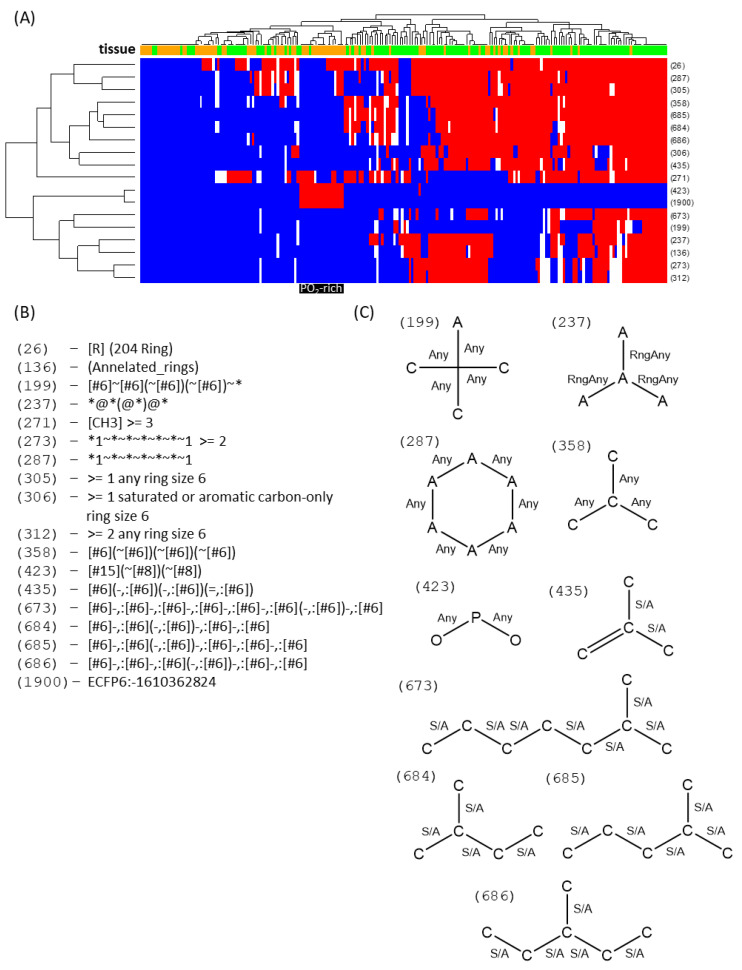
Fingerprint heatmaps of non-polar DAFs. A heatmap (**A**) is shown of the DAFs (columns) that had a fingerprint vector assigned, colored by tissue (green: leaf; orange: pitcher) on the top band. Only the enriched fingerprints (rows) are shown, named by CSI-FingerID relative index position (**A**). Based on posterior probabilities, the fingerprints were determined to be absent (blue), present (red), or uncertain (white). A cluster of DAFs almost exclusively accumulated in pitchers is highlighted in black, with the enriched fingerprints being described in (**B**) and, if graphical representation is possible, in (**C**). Any means it can be any kind of bond, RngAny means the bond is in a ring (of any kind), S/A means it is a single bond that can be anywhere (within a ring or not).

Strikingly, pitcher DAFs have an increased presence of phosphate groups (Figure 5). They also mostly lack tertiary and quaternary carbons and rings, which would point at acyl lipids and phospholipids as those lipids in pitchers that best differentiate them compared with leaves. Accordingly, leaf DAFs have a distinctive annulated ring structure, along with fingerprints of at least two six-carbon rings, ternary carbons and branched fatty acyl chains, all typical fingerprints of sterol lipids. Indeed, analyzing the heatmap of the selected vectors (Figure 5) it can be seen that the right-most clusters, with most of the leaf DAFs, show typical sterol fingerprints. In contrast, the left-most clusters, with the majority of the pitcher DAFs, have at most one ring. In addition, this cluster harbors the prominent PO_2_-containing cluster, consisting almost entirely of pitcher DAFs. 

**Figure 6:**
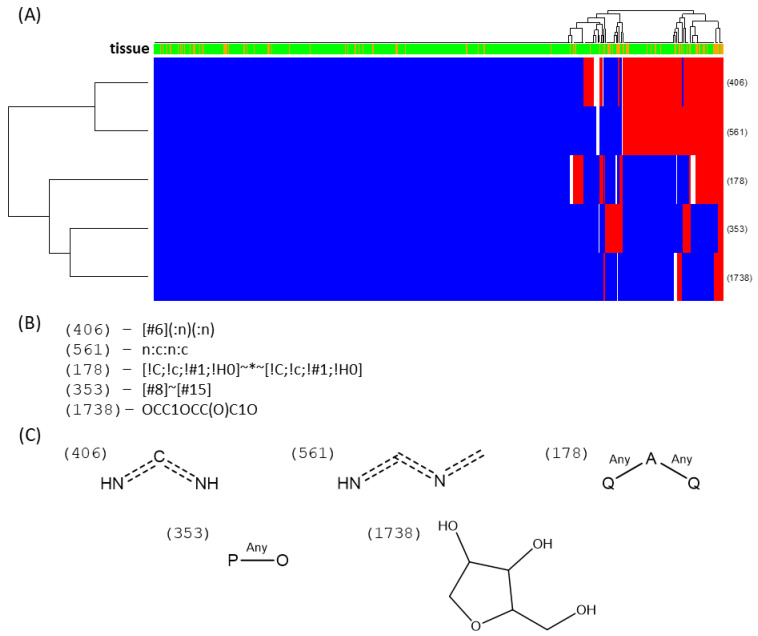
Fingerprint heatmaps of polar DAFs. A heatmap (**A**) is shown of the DAFs (columns) that had a fingerprint vector assigned, with blue cells being present, red being absent, and white being uncertain fingerprints. Given the nature of the sample, being mostly leaf DAFs, only the positive fingerprints enriched in pitcher and absent in leaf are shown. These fingerprints are described in (**B**) and the graphical approximation of their substructure in (**C**). It is important to note that the right-most cluster is unusually enriched in pitcher DAFs, with a high number of positive assignations of most of the selected fingerprints.

Concerning the fingerprints of the polar extracts, there are many more DAFs in leaves than in pitchers. Because structural variability is strikingly higher in polar compounds, interpretation is less straightforward. However, pitcher DAFs are seemingly enriched in compounds with heteroatoms, such as nitrogen or phosphate, and pentose fingerprints. Some diimines are found naturally in purines and ureides—both soluble molecule families that have a high nitrogen load. Given that there are five times more DAFs with fingerprints in leaf than in pitcher, not many characteristic fingerprints can be robustly assigned to be leaf-specific. Nevertheless, one of the main DAFs found in leaf blades, which appears to be 32 times higher in leaf blades than in pitchers, has been tentatively identified as the naphthoquinone plumbagin. In sum, in the corresponding fingerprint heatmap (Figure 6) the enrichment is not as clear cut as in the lipids, given the low abundance of pitcher DAFs. However, it is still noticeable that the right-most cluster concentrates almost exclusively pitcher DAFs: of the 11 DAFs simultaneously having four of these five fingerprints, only one is from leaf. Only one of these compounds had a biologically relevant database hit, resembling a uridine bisphosphate. In addition, interestingly, only five out of the 16 DAFs with a pentose fingerprint are accumulated in leaf.

### 2.3. Differences in Pitcher Due to Feeding Status

As the PCA suggested that only the polar extract of pitchers had a difference depending on feeding status, and to avoid interference with external variance, a one-way ANOVA was performed specifically in the polar extract of fed and non-fed pitchers. Thus, we found 73 DAFs due to the feeding status, with 27 features accumulating in fed pitchers, and 46 accumulating in non-fed pitchers (Figure S6). Unlike the above-mentioned examples, fold changes appear to be balanced, although the features accumulating in fed pitchers appear to have a higher *m*/*z* than those in non-fed pitchers (Figure S7). Notably, almost all of the DAFs (69 out of 73) are present only in the concentrated extract, and even there with low intensity. 

Since most compounds do not have fragmentation due to low intensity, the full pipeline of SIRIUS+CSI-FingerID was followed, and the candidate list was manually curated. The results are shown in Table S3, where it can be seen that only 11 DAFs had a fragmentation pattern that allowed structural interpretation. Although the largest DAF-containing group is the one of non-fed pitchers (46 DAFs), only four features have assignations. Interestingly, three are nitrogenated: a putative nitrogenated heptose (C_7_H_15_NO_9_), an unidentified, densely nitrogenated compound (C_13_H_17_N_9_O_12_), and a third that appears to be a nucleotide phosphate with an either cyclic (C_10_H_17_N_4_O_7_P) or acyclic (C_10_H_15_N_4_O_6_P) attachment. As for the fed pitcher, seven DAFs were identified, four likely to be phenolic compounds and three nitrogenated compounds. The phenolics were likely three simple phenolics (C_10_H_10_O_3,_ C_17_H_22_O_8_, and C_13_H_14_O_11_, the latter two glycosylated) and a flavonoid (C_17_H_14_O_7_). The nitrogenated compounds had no hits in biologically relevant databases, only in PubChem; of those, two were compounds with four nitrogen atoms (C_22_H_24_N_4_O_7_ and C_27_H_18_N_4_O_6_) with very similar fingerprints, with more than two aromatic rings and nitrogen atoms in heterocycles, and the remaining one (C_14_H_16_NO_5_) had a single aromatic ring and a single nitrogen. 

## 3. Discussion

Many low-molecular-weight compounds identified so far in carnivorous plants are volatile compounds suggested to be involved in prey attraction [19,33]. For instance, in *N. rafflesiana*, more than 50 volatiles have been found [34]. Less information is available for non-volatile compounds. Thus, we performed an untargeted metabolomics approach to determine which compounds might be related to carnivory in the metabolism of *Nepenthes x ventrata,* used here as a model plant. Two different questions have been addressed; first, we wanted to see whether or not the leaf blade and the pitcher contain different tissue- and function-specific metabolite patterns; second, we looked for differences in the tissues before and after insect feeding. This is the first study where a metabolomic profiling of the carnivory process in the genus *Nepenthes* is performed. Due to the technical design of this untargeted metabolomics work, the vast majority of primary metabolites fall inside the exclusion range for fragmentation (50–150 *m*/*z*); therefore, no meaningful assignation of identity or fingerprints could be performed on primary metabolites.

### 3.1. Metabolite Differences in Nepenthes Tissues: Leaf Blade vs. Pitcher

Overall, the number of features observed in leaves was much higher compared with pitcher tissue. In particular, there is a clear trend for the presence of polar compounds with *m*/*z* > 300 and of non-polar compounds with *m*/*z* > 400 in leaves. In addition, more over-accumulated features were found in leaves, with higher fold changes compared to pitcher. This means that both metabolite levels and diversity are lower in pitchers. 

In the non-polar phase, the DAFs that best discriminate between pitcher and leaf are very likely acyl lipids and phospholipids, which are preferentially found in pitchers, and sterol derivatives, which are preferentially accumulated in leaves. The different membrane composition of these two tissues may be reflective of the differing functions. Sterols affect membrane fluidity and permeability, making the membranes more rigid, and are considered membrane reinforcers [35]. In addition, sterols are critical for the formation of lipid “rafts”, which regulate biological processes such as signaling and transport across the membrane [36]. In *Nepenthes*, first, nutrient uptake from the pitcher fluid is performed by the bi-functional glands localized inside the pitcher. Besides carriers, clathrin-mediated endocytosis is involved in this process [37]. Specific for the vesicles of the clathrin-mediated pathway are phospholipids, favoring vesicle formation in contrast to sterols [38]. This might be another point that explains the different distribution of lipophilic metabolites in pitchers and leaves. In addition, a unique feature of *Nepenthes* pitchers is the waxy coating of the inner part of the pitcher, making it slippy for any prey trying to escape. This might also explain the difference in lipophilic metabolites in the pitcher compared with the leaf.

Interestingly, there is a family of polar compounds that simultaneously have a methylene-interrupted heteroatom, diimine-like structure (*~N=C=N~* and *~N=C=N=C-*), and phosphate and pentose fingerprints, and are exclusive to pitchers (10 out of the 11 DAFs with at least four of the five fingerprints). This finding was surprising as the carnivorous plants actually are limited in nitrogen and phosphate, and none of these DAFs are changing significantly due to feeding status. Nevertheless, since pitchers need to be ready for catching and digesting prey, they might be active in transport of both phosphate- and nitrogen-containing compounds. The presence of nucleotide phosphates supports the view at the pitcher as an active tissue ready to start de-novo synthesis of all necessary biosynthetic pathways. As long as no prey or not enough prey has been caught, even the pitcher must be seen as a sink tissue, and transport can occur in any direction. The putative nitrogen- and phosphate-containing glycosylated compounds are not present in biological databases and may hold valuable information on nitrogen and phosphate transport. The nature of these compounds, which might be mobile within the plant, is still an open question. Nitrogenous bases, like ureides, are well known to undergo long-distance transport in rhizobia–legume symbioses [39] as well as in non-nodulated plants [40]. Interestingly, the final enzymatic step to release ammonia from ureides is catalyzed by a urease. Its presence and activity were recently demonstrated for *Nepenthes* and other carnivorous plants [41]. Whether or not this scenario mirrors the nitrogen translocation and distribution that occurs in *Nepenthes* remains to be elucidated.

### 3.2. Insect Feeding Causes Changes in Polar Metabolite Pattern in Pitchers

In order to better understand the dynamics of the metabolic processes of carnivory in *Nepenthes* plants, immediately after opening, the pitchers were fed with fruit flies or not fed for 72 h. Results of the MS-based untargeted metabolomics analysis determined small but significant changes only in the pitcher tissue and, moreover, only in the fraction containing the polar metabolites. No significant changes in the leaf blade and no changes in the pitchers’ non-polar metabolites were found as a result of feeding. Nevertheless, there was a trend showing that fed pitchers accumulated more compounds with higher molecular weight compared with non-fed pitchers, indicating a modulated, increased metabolic activity. Without knowing the exact structures of the compounds, the ecological relevance of changes in metabolite composition remains speculative. It might be due to higher physiological activities, in the sense that mobile compounds are built which can more easily be distributed within the plant or that the pitcher tissue needs to be more defended against detrimental organisms showing up together with caught prey. This would explain an increase in, for example, some phenolic compounds. For example, in our experiment, the fed pitchers were found having an around four times higher concentration of a flavonoid-related feature (c_331.0809-12.16; C_17_H_14_O_7_; Table S3) compared with non-fed pitchers. It is also suggested that *Nepenthes* is a slowly digesting plant [42]. For example, prey-initiated induction of digestive enzymes such as the protease nepenthesin can take days [43]. Thus, it is conceivable that the selected 72 h of prey digestion were not sufficient to detect more induced metabolites, qualitatively or quantitatively. Following this thread, it may also explain why no effect of feeding was found in the leaf blades. Experiments with *N. hemsleyana*, a coprophagous *Nepenthes* species that does not catch prey any more but feeds on bat feces [44], showed that upon ^15^N-enriched urea application into pitchers, after only four days, ^15^N was significantly detectable in protein fractions of leaf blades [41]. 

These data suggest the lipid composition of pitcher appears to favor vesicle formation, while leaf blade lipids promote rafts and membrane rigidity; pitcher-specific DAFs contain nitrogen and phosphorus, with typical fingerprints of molecules known to undergo long-distance transport; and changes in leaf and pitcher features are weak due to feeding status. We may further speculate that prey-derived nutrients are taken up via vesicles in the pitcher, further degraded, fixed in organic N- and P-rich compounds, and eventually systemically distributed, thereby passing the proximal leaf blades. This is supported by research showing that developing leaves incorporated a higher level of ^15^N, being preferentially supplied compared with a leaf that carries a fully developed pitcher [45]. Additional future experiments with different time points of harvesting may provide more insight into the dynamics of prey-induced changes in the *Nepenthes* metabolome in different tissues. However, as carnivorous plants mainly hunt for nitrogen and phosphate, it was not surprising to find prey-induced metabolite changes in the fraction containing polar, water-soluble compounds. 

LC-MS-based metabolomics is a powerful tool for assessing chemical diversity in an un-biased manner, and is particularly useful for characterizing non-model plants, for which available data is scarce. However, the very nature of understudied plants complicates interpretation of the results, as most methods of putative identification rely heavily on databases, suffering greatly from popularity bias, and require manual curation, hindering analysis of systemic changes, such as those in pools of metabolites. Cheminformatics has long been used to extract information from large databases in an automated manner, but usually requires the existence of a chemical structure. We used a cheminformatics-aided metabolomics approach for characterizing the carnivorous plant *N. x ventrata*, using CSI-FingerID [31] fingerprint vectors directly, entirely bypassing structure assignation, the weakest link in the metabolomics pipeline. This minimizes false positives, and produces a robust, evidence-based approach for exploring systemic changes in metabolites. 

In order to elucidate the real structures of the numerous compounds, further analyses are necessary, such as NMR. However, the compounds we found occur at low abundance, and this makes it extremely difficult to isolate enough material for analysis. However, the methods employed in the present study highlight general tissue-specific metabolites and their changes upon prey digestion. 

Nevertheless, the fact that many features could not be identified in biologically relevant databases highlights the need to characterize non-model plant species to increase our knowledge of chemical diversity and find still-unknown compounds, which might be biologically or pharmaceutically relevant. In particular, *Nepenthes* species are well known in traditional medicine. Various reports are available describing curative effects of extracts from different *Nepenthes* species and tissues on diseases, for example, on cough, fever, hypertension, urinary system infections [46], malaria [47,48], asthma, pain [48]; *Staphylococcus* infection [49], celiac disease [50], and recently on different kinds of oral cancer cells [51]. Thus, further work on the isolation and structure elucidation of *Nepenthes* metabolites as well as the analysis of their putative pharmaceutical uses seems promising in order to find new structures and therapeutics.

In conclusion, the studied *Nepenthes x ventrata* plant contains a huge variety of different metabolites. We focused on MS-based and data mining approaches to visualize the metabolic differences between leaf and pitcher tissues, and between fed and un-fed plants. Leaf metamorphosis into pitchers and leaf blades generated new tissues that are different in function, which is also clearly represented in their respective DAFs. Surprisingly, insect prey feeding has a much smaller impact on the measured metabolites. Cheminformatics approaches suggest the presence of many structurally unknown compounds which might be of therapeutic interest, bearing in mind that *Nepenthes* species have been long used in traditional medicine. Further research should be carried out addressing the remaining questions of metabolite identification, biosynthetic pathways and the ecological relevance of *Nepenthes* metabolites.

## 4. Materials and Methods

### 4.1. Plant Material, Treatment, and Sampling

We used the natural hybrid *Nepenthes x ventrata* (*N. alata x N. ventricosa*) as a model organism. *N. x ventrata* plants were grown in the greenhouse of the MPI for Chemical Ecology at 21–23 °C, 50–60% relative humidity and a 16/8 h light/dark photoperiod. To avoid contamination, still-closed pitchers were covered with a mesh. Once the pitchers opened, they were left untreated for controls or prey degradation was induced by adding 30 wild-type *Drosophila melanogaster*, representing ca. 31 mg fresh weight. Individual pitchers represent independent biological replicates from different plants. After 72 h, pitchers were emptied, i.e., the digestive fluid with or without the remains of fruit flies was discarded, and subsequently rinsed 3 times with sterile distilled water. Next, both the tissue from the glandular zone (lower third part of the pitcher) and the related leaf blade were dissected and sampled in 50-mL Falcon tubes and immediately frozen in liquid nitrogen. The plant material was finely ground in liquid nitrogen using a mortar and pestle. Then, ground material was stored in screw-cap Eppendorf tubes and stored at −80 °C until further processing.

### 4.2. Metabolomic Extraction

Altogether, 28 individual samples were examined—7 *D. melanogaster*-treated and 7 untreated pitchers—and their corresponding leaf blades harvested after 72 h. Samples were extracted following a procedure derived from [52,53] with some modifications. In short, double extractions of 100 mg FW tissue powder were performed in 2-mL Eppendorf tubes at room temperature, using 500 µL MeOH:ammonium acetate buffer (pH 4.8). Therefore, after 5 min shaking, a 15 min sonication in water bath followed (3× for 5 min and 3 min resting in between). Extracts were centrifuged at 20,000× *g* for 10 min. Clear supernatants were combined and filtrated using a PTFE syringe filter (hydrophilic 0.22 µm pores, 13 mm diameter, Fisherbrand, Cat.# 15161499, Fisher Scientific, Schwerte, Germany). This extract was diluted 1:10 with 75% MeOH and further analyzed. 

### 4.3. Lipidomics Extraction

Here, altogether 30 individual samples were examined: 5 non-treated control pitchers and leaf blades were taken directly after pitcher opening at 0 h; 5 *D. melanogaster*-treated and 5 untreated pitchers and their corresponding leaf blades taken after 72 h. Each sample represents an independent biological replicate. Extractions were done following a procedure derived from Matyash et al. (2008) [54] and Chen et al. (2013) [55] with some modifications. All steps were performed in glass test tubes and kept at room temperature. In short, an adjusted volume of methanol was added to 100 mg FW of tissue powder, based on a ratio of 150:1 *v*/*w* DW. Milli-Q water was added to a final ratio of 3:1 MeOH:H_2_O, taking the water content (87%) of the tissues into consideration, which was determined before. Next, samples were vortexed followed by 5 min sonication in a water bath (5× for 1 min and 1 min resting in between). Thereafter, methyl-*tert*-butyl ether (MTBE) was added to achieve a ratio of 10:3:1 (MTBE:MeOH:H_2_O). Samples were vortexed again, sonicated as described and shaken at 100 rpm for 1 h. Afterwards, milli-Q water was added to reach a total ratio of 20:6:7 (MTBE:MeOH:H_2_O). Samples were vortexed, sonicated as previously described, and shaken for 10 min. To separate them into two phases, samples were centrifuged at 100× *g* for 20 min. The organic phase was recovered, while the aqueous phase was extracted again in 2 mL, keeping the ratio of MTBE:MeOH (20:6:7). Both organic phases were combined and evaporated under vacuum at 45 °C. The dry aqueous and organic samples were resuspended in acetonitrile:isopropanol (50:50) to a concentration equivalent to 1 g/L DW and filtrated using a PTFE syringe filter. This extract was diluted 1:10 with acetonitrile:isopropanol (50:50) and further analyzed.

### 4.4. Metabolic Profiling Using HPLC-qToF-MS

Samples were analyzed using an Elute LC system (Bruker Daltonik, Bremen, Germany) coupled via ESI to a Maxis II q-TOF (Bruker Daltonik, Bremen, Germany). Polar compounds were separated using a Kinetex^®^ XB-C18 column (100 × 2.1 mm, 2.6 µm, 100 Å; Phenomenex, Aschaffenburg, Germany) at 40 °C with a gradient from water to acetonitrile, both modified with 0.1% formic acid, according to [52] with minor modifications. Namely, there was a flow of 0.2 mL/min, a linear gradient from 5% to 75% acetonitrile over 20 min, increased linearly to 95% acetonitrile over 5 min, followed by a 5-min equilibration at the initial conditions. Non-polar compounds were separated using a Luna^®^ Omega PS C18 column (150 × 2.1 mm, 3 µm, 100 Å; Phenomenex, Aschaffenburg, Germany) at 50 °C. Mobile phase A was a mixture of water and acetonitrile (4:1 *v*/*v*) and mobile phase B was an isopropanol:acetonitrile mixture (9:1 *v*/*v*); both phases were modified to a final concentration of 10 mM ammonium acetate and 0.1% formic acid. The gradient was as previously published [56] with minor modifications: at a flow of 0.2 mL/min, a linear increase from 40% B to 45% B in 2 min, then to 55% B in 8 min, followed by an immediate step increase to 70% B, then a linear increase to 99% B in 10 min, holding at 99% B for 5 min, and finally returning to the initial conditions for 5 min. For analysis of the extracts, 5 µL of a 10-fold dilution was injected, and, for the polar extracts, a second batch of 5 µL of concentrated extract was injected. Injections in each of these three batches were randomized, with 5 evenly interleaved quality control injections of pooled samples, preceded by 4 “dummy” injections of pooled quality control samples to passivate the column, which was extensively washed after each batch. Analyses of the quality control samples are shown in Figures S8–S10.

Acquisition of MS data was done using the same conditions for both polar and non-polar compounds. Ionization was performed via pneumatic-assisted electrospray ionization in positive mode (ESI+) with a capillary voltage of 4.5 kV and an end plate offset of 500 V; a nebulizer pressure of 3 bar was used, with nitrogen at 350 °C and a flow of 12 L/min as the drying gas. Acquisition was done at 12 Hz following a mass range from 50 to 1000 *m*/*z*, with data-dependent MS/MS and an active exclusion window of 0.2 min, a reconsideration threshold of 1.8-fold change, and an exclusion range of 50–150 *m*/*z*. Fragmentation was triggered on an absolute threshold of 400 and acquired on the most intense peaks using a target intensity of 20,000 counts, with MS/MS spectra acquisition between 12 and 20 Hz, and limited to a total cycle time range of 0.5 s. Collision energy was determined automatically by the software depending on *m*/*z* value. At the beginning of each run, an injection of 20 µL of a sodium formate–isopropanol solution was performed in the dead volume of the injection, and the *m*/*z* values were re-calibrated using the expected cluster ion *m*/*z* values.

### 4.5. Feature Detection

Peak detection was done using Metaboscape software (Bruker Daltonik, Bremen, Germany) with the T-Rex 3D algorithm for qTOF data. For the non-polar runs, parameters for detection were an intensity threshold of 500 with a minimum of 7 spectra, and features were kept if they were detected in at least 3 replicates of the same treatment, tissue and time (60% of n). Adducts of [M+H]^+^, [M+Na]^+^, [M+K]^+^, and [M+NH_4_]^+^ were grouped as a single feature if they had an EIC correlation of 0.8. For the polar runs, the intensity threshold was set to 1000, the features were kept if detected in at least 5 replicates of the same treatment and tissue (70% of n), and adducts were grouped in the same manner, only excluding the ammonium adduct, which was not expected in the polar runs. 

### 4.6. Spectral Analysis

Proprietary MS Bruker files were re-calibrated with cluster ions of sodium formate in the dead-volume injection time and converted to mzXML [57,58,59] using Bruker DataAnalyst software (Bruker Daltronik, Bremen, Germany). Access to the raw data in mzXML files was done in R with the aid of the *mzR* library [60]. MS/MS data was extracted for selected features using an in-house built code that searched in all samples for fragmentation events triggered in a window of 0.5 min within the feature retention time (RT). To avoid misassignation of closely eluting isobaric compounds within the RT window, the maximum of intensity in the MS1 extracted ion chromatogram (XIC) of the feature *m*/*z* (with 5 ppm error) that was closest to the feature RT was searched. Only contiguous peaks decreasing in intensity from the previous point in the MS1 XIC and with intensity higher than 10% of the maximum were kept. The new RT window was determined by the time in the first and last events. Within this new RT window, all fragmentation events whose parent ions matched the feature *m*/*z* within a 5 ppm error were stored. The fragmentation events of the most abundant 5 (non-polar) and 7 (polar) peaks for each feature adduct were merged using previously published in-house binning algorithm [61], and saved as MASCOT generic format (MGF) files. 

Candidate structures and database-independent fingerprint vectors were obtained by loading the above-mentioned MGF files into the SIRIUS [28,29,30] and CSI-FingerID [31] pipeline. Candidate structures for the DAFs of fed and non-fed pitchers were obtained by searching the top hit of CSI-FingerID in all databases and manually curating the results; for all the other analyses, fingerprint vectors of the top 10 candidates of all predicted formulas were exported and loaded in R. When more than one adduct was present in a feature, only the formulas that matched the formulas of the adducts were kept. Then, only fingerprints that explained more than 3 peaks and more than one third of the intensity were kept. The final selection of the fingerprint vectors was made by collapsing all the adducts per feature, only keeping the fingerprint vectors corresponding to the top-scoring candidate and those that were less than 30% different. Fingerprints were assigned as present if the highest posterior probability of fingerprint vectors and adducts was greater than 0.75, as absent if the lowest posterior probability was less than 0.25, and as uncertain otherwise. Enrichment for presence and absence were calculated via a hypergeometric test, with uncertain assignations not being considered in the probability calculations as either hits or fails. The p-values of the hypergeometric tests were corrected for multiple testing. 

### 4.7. Statistical Analysis

All statistical analyses were performed using the R 3.6.1 *base* package [62] and graphics using a combination of the *ggplot2* [63] and *gplots* [64] libraries, unless otherwise specified. Analysis of polar and non-polar fractions was done separately, given the nature of the experiments. Since the maximum signal-to-noise ratio was assumed to be 1/3, the zeroes in the matrices were replaced by their respective minimum measured area, divided by three, and then log_10_-transformed. The resulting matrices, estimated as Normal by Q-Q plots, were used for ANOVAs. For principal component analysis, these log_10_-transformed matrices were z-scaled by subtracting the mean and dividing by the standard deviation in a feature-wise manner. For the non-polar analysis, a two-way ANOVA was done on samples after 72 h, taking tissue and treatment as factors, and blocking by extraction batch. Since no difference was found by treatment, the 0 h control was added to analysis discriminating tissue, blocking by all other variables. For the polar analysis, a two-way ANOVA was done on the concatenated matrix of concentrated and diluted injections, taking tissue and treatment as factors. The features were de-duplicated only after statistical testing and false discovery rate correction, and this deduplication was only performed on significantly different peaks. Features were considered duplicated if they shared the same *m*/*z* (within 10 ppm or an absolute 0.0025 difference) and retention time (within 0.15 min) and were not detected as different features in either the concentrated or diluted injections. That is, if 3 (significantly different) features were detected in the concentrated batch within that window (10 ppm, 0.15 min), and 2 (significantly different) features were detected in the diluted sample, the deduplication would keep all 3 (significantly different) features in the concentrated sample because, even when they share *m*/*z* and RT, they were detected as different features by MetaboScape. This is a conservative approach for calculating both FDR and fold change. All statistical testing was controlled for multiple testing by Benjamini and Hochberg’s (1995) [65] false discovery rate correction.

## Supplementary Materials

The following are available online at https://www.mdpi.com/1422-0067/21/12/4376/s1, Figure S1: PCA Scores vs. *m*/*z*; Figure S2: Heatmap of polar DAFs; Figure S3: Heatmap of non-polar DAFs; Figure S4: The *m*/*z* plots; Figure S5: Fold-change density plot; Figure S6: Heatmap of polar DAFs in pitchers due to feeding status; Figure S7: Feeding fold-change density plots; Figure S8: Quality control injections in the lipidomics experiment; Figure S9: Quality control injections in the polar experiment, injecting the raw extracts; Figure S10: Quality control injections in the polar experiment, injecting the ten-fold diluted extracts; Table S1: Polar fingerprints; Table S2: Non-polar fingerprints; Table S3: Features. Raw data was deposited in Metabolights Study MTBLS1783, as well as in the EDMOND database (DOI: https://dx.doi.org/10.17617/3.42).
