# Supplementary material for: Metabolomics Analysis Reveals Tissue-Specific Metabolite Compositions in Leaf Blade and Traps of Carnivorous Nepenthes Plants
Source: Int J Mol Sci. 2020 Jun 19;21(12):4376. doi: 10.3390/ijms21124376 (PMC7352528; doi:10.3390/ijms21124376)
Supplement: Supplementary file 1 [file ijms-21-04376-s001.zip › ijms-823268-supplementary/Supplementary Materials.docx]

**SUPPLEMENTARY MATERIALS:**

**Metabolomics analysis reveals tissue specific metabolite compositions in leaf blade and traps of carnivorous *Nepenthes* plants**

Alberto Davila-Lara, Carlos E. Rodriguez Lopez, Sarah E. O’Connor, Axel Mithöfer

**7 Supplementary Figures**

**3 Supplementary Tables (Excel file)**


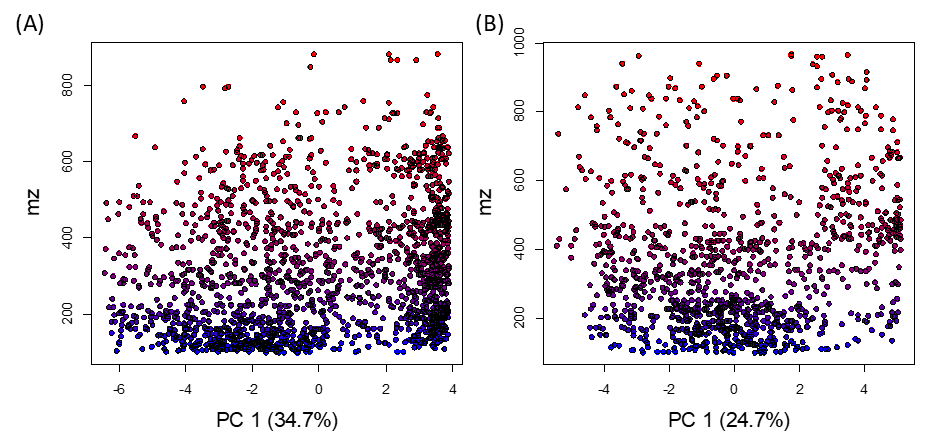


**Supplementary Figure 1.** PCA Scores vs *m/z*. All features for polar (A) and non-polar (B) extracts are shown, plotted by their main component score and the *m/z*. Circles are colored by *m/z*, from low (blue) to high (red) *m/z* value of the feature, to aid visualization.


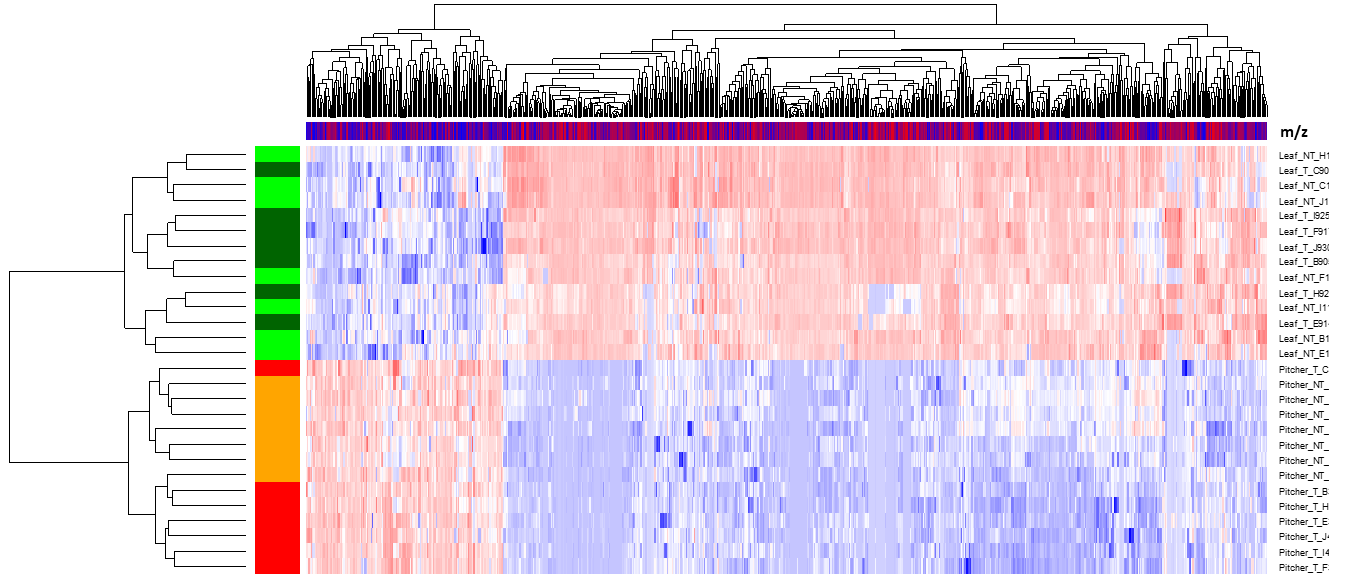


**Supplementary Figure 2.** Heatmap of polar DAFs. A heatmap for all DAFs (FDR<0.01) of the polar extracts, with low abundance being blue and high abundance in red. The band on the top is colored by *m/z* from low (blue) to high (red) *m/z*, and the left band by tissue and feeding status: dark green and light green being fed and not-fed leaves; and red and orange, fed and not fed pitcher, respectively.

**
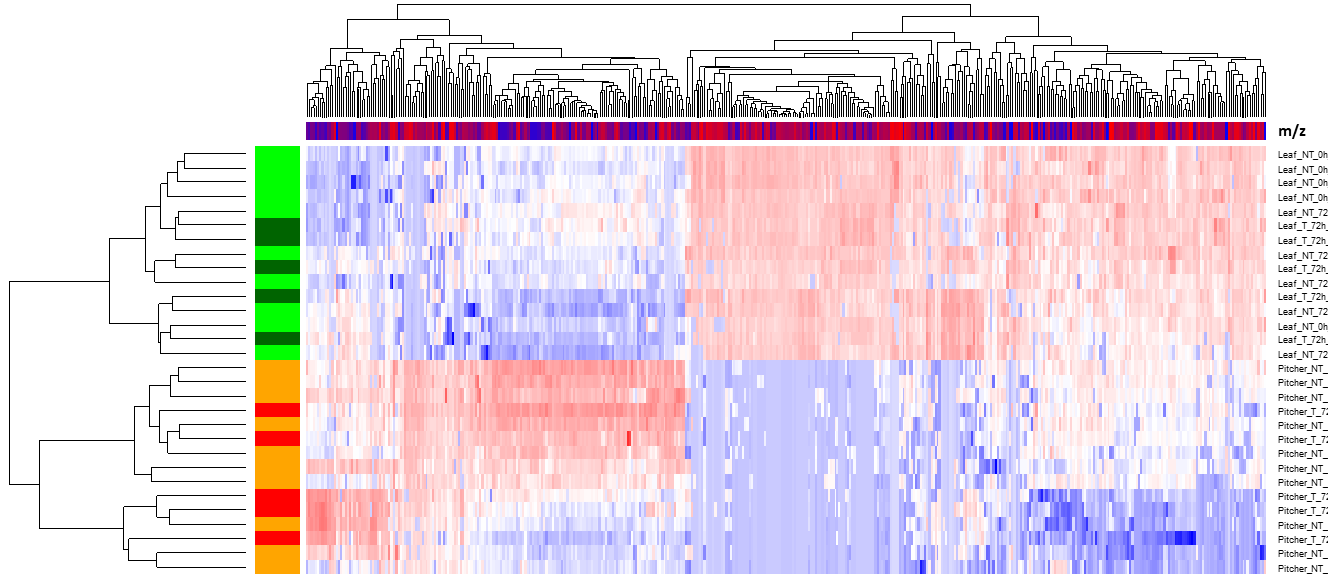
**

**Supplementary Figure 3.** Heatmap of non-polar DAFs. A heatmap for all DAFs (FDR<0.01) of the non-polar extracts, with low abundance being blue and high abundance in red. The band on the top is colored by *m/z* from low (blue) to high (red) *m/z*, and the left band by tissue and feeding status: dark green and light green being fed and not-fed leaves; and red and orange, fed and not fed pitcher.

**
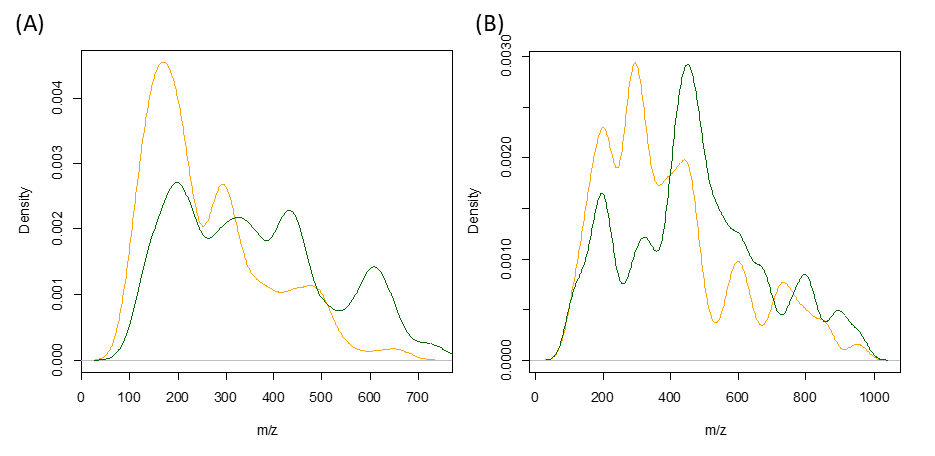
**

**Supplementary Figure 4.** The *m/z* density plots. A plot of kernel density estimates for all polar (A) and non-polar (B) DAFs’ (FDR<0.01) measured *m/z*, colored by tissue: Orange being all fed and not fed pitcher, and dark green all fed and not fed leaves. The lines depict the distribution of *m/z* values in the corresponding samples.


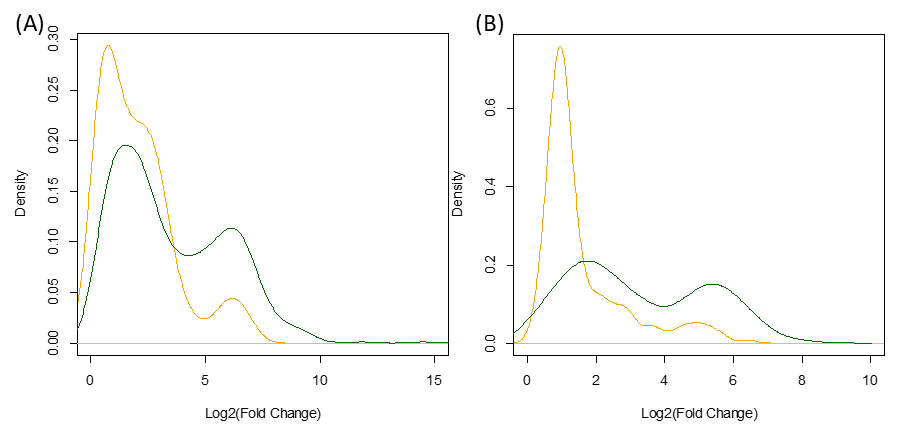


**Supplementary Figure 5.** Fold-change density plots. A plot of kernel density estimates for all polar (A) and non-polar (B) DAFs’ (FDR<0.01) absolute log_2_ fold-change value. In orange, pitcher, and in dark green, all leaves fold-change distributions.


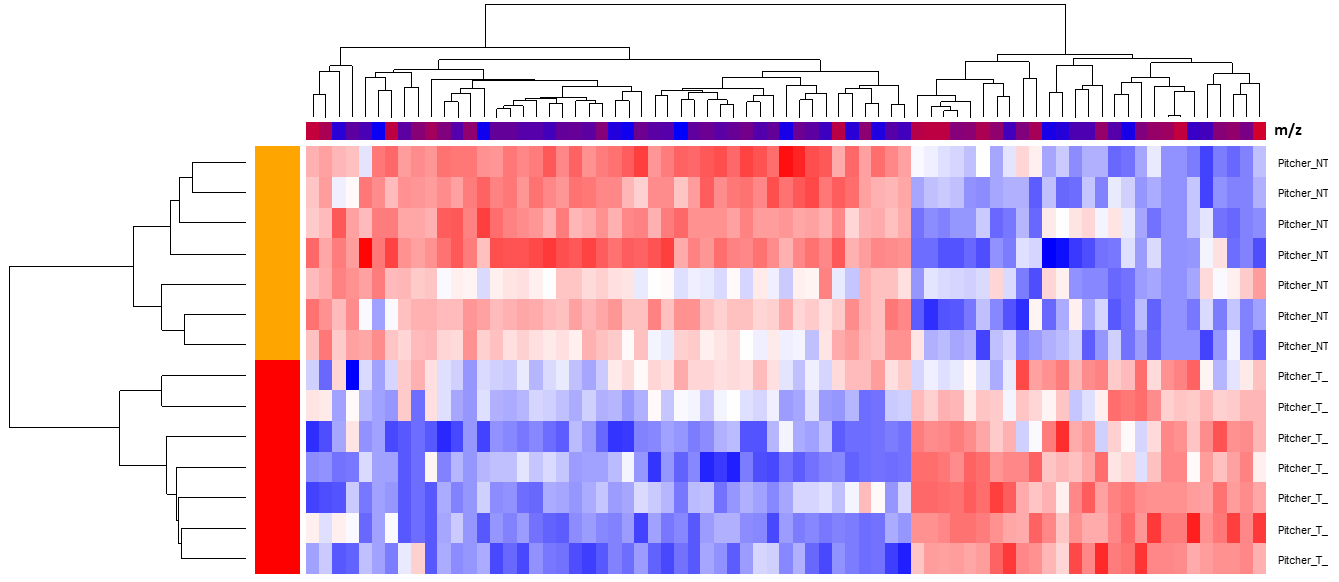


**Supplementary Figure 6**. Heatmap of polar DAFs in pitchers due to feeding status. A heatmap for all DAFs (FDR<0.05) of the polar extracts of pitchers, with low signal being blue and high signal in red. The band on the top is colored by *m/z* from low (blue) to high (red) *m/z*, and the left band representing fed (red) and not-fed (orange) pitcher.

**Supplementary Figure 7**. Feeding fold-change density plots. A plot of kernel density estimates for *m/z* of polar DAFs’ (FDR<0.05) due to feeding status of pitchers. In red, fed, and in orange non-fed pitcher *m/z* distributions.
